# Relevant pharmacokinetics, bioavailability, and bioequivalence studies on Chlorpheniramine maleate (various species): a review

**DOI:** 10.3389/fphar.2025.1737690

**Published:** 2025-12-12

**Authors:** César Alas-Pineda, Dennis J. Pavón-Varela, Kristhel Gaitán-Zambrano, Gustavo Ferrer

**Affiliations:** 1 Department of Research & Development, Moxie Health Group, Florida, FL, United States; 2 Department of Pulmonary and Critical Care Medicine, Aventura Hospital and Medical Center, Aventura, FL, United States

**Keywords:** Chlorpheniramine, pharmacokinetics, intranasal drug delivery, bioavailability, drug repurposing, mucosal transport, H1 antihistamines

## Abstract

**Introduction:**

Chlorpheniramine maleate (CPM) is a first-generation H1-antihistamine widely used for allergic conditions, yet its pharmacokinetic (PK) and bioavailability profiles across species remain poorly characterized. Understanding interspecies variability is critical for translational applications and the development of novel formulations. This review aims to summarize and critically evaluate the pharmacokinetics, bioavailability, species-specific behavior, mechanistic insights, and formulation-dependent variability of CPM, with emphasis on intranasal and buccal administration routes and their translational potential.

**Methods:**

We conducted a scoping review in accordance with PRISMA-ScR 2018 Guidelines on studies assessing CPM pharmacokinetics in hu-mans and animal models. The identification phases consisted of keyword terms mesh in PubMed: Search 1: Chlorpheniramine Bioavailability (n = 38), Search 2: Chlorpheniramine Bioequivalency (n = 14), and Search 3: Intranasal Chlorpheniramine (n = 54). Repeated or irrelevant studies were excluded, with a total of 22 studies analyzed, from which 13 are included in the final report.

**Results:**

CPM exhibits moderate oral bioavailability (25%–50%) and extensive tissue distribution, with a long elimination half-life (∼20 h). Intranasal and buccal routes demonstrate faster absorption and partial hepatic bypass. Bioequivalence studies reveal significant formulation-dependent variability, influenced by excipient design, release profiles, and stereochemistry.

**Conclusion:**

CPM remains a pharmacologically valuable molecule with underexplored delivery routes and applications. Standardization of formulations, population-specific pharmacokinetics, and further trials are warranted to unlock the full therapeutic potential of this approach beyond classical allergy treatment.

## 1 Introduction

Chlorpheniramine Maleate (CPM) is a first-generation H1-antihistamine within the alkylamine class. It acts primarily as an inverse agonist at the H1 histamine receptor, thereby reducing the physiological effects of histamine release associated with allergic conditions (Athanikar and Chiou, 1979). Early pharmacological and pharmacokinetic investigations established its absorption and tissue distribution in both animals and humans (Sakurai et al., 1992; Tung et al., 2001; Rumore, 1984). Due to its lipophilic nature and ability to cross the blood-brain barrier, CPM also exerts mild sedative and anticholinergic effects (Rizvi et al., 2024). Importantly, many of these emerging indications depend on achieving rapid and targeted drug concentrations in mucosal tissues, where CPM’s absorption characteristics differ significantly by route. For antiviral and anti-inflammatory applications, the speed of delivery and local tissue exposure may determine clinical effectiveness, highlighting the need to integrate pharmacokinetic knowledge with therapeutic repurposing efforts.

The molecule exhibits a tertiary amine structure with p-chlorophenyl and pyridyl substitutions, contributing to both its receptor affinity and pharmacokinetic profile (Rizvi et al., 2024). Studies in rats demonstrated stereoselective pharmacokinetics of CPM enantiomers (Hiep et al., 2000), while investigations in cattle provided insights into its metabolism and systemic exposure in large animals (Chou and Donovan, 1997; Kandimalla and Donovan, 2005).

In addition to its use in allergy-related symptoms, CPM has demonstrated serotonin reuptake inhibition and potential antiviral effects, including against influenza and SARS-CoV-2 (Rizvi et al., 2024). These effects have sparked interest in repositioning CPM for new therapeutic indications, such as viral infections and neuropsychiatric disorders (Rizvi et al., 2024). Although primarily used in over the counter (OTC) treatments for coughs and colds, various studies discuss a wide range of CPM’s clinical uses, including the treatment of asthma, plasma cell gingivitis, chronic urticaria, and depression, among others (Rizvi et al., 2024).

Interest in non-oral routes of CPM delivery predates modern antiviral research. Early vascular and absorption studies in dogs and humans demonstrated that intranasal CPM exhibits rapid uptake and pronounced mucosal effects, providing the foundation for the current resurgence of interest in nasal formulations. Intranasal administration of CPM was evaluated in canine and human models during the 1980s, showing significant vascular effects on the nasal mucosa and supporting its potential for local and systemic delivery (Ichimura and Jackson, 1985; Lung and Wang, 1987). More recently, new formulations of intranasal CPM have been developed and studied in the context of viral respiratory infections, including COVID-19 (Torres et al., 2021; Ferrer et al., 2025; Valerio-Pascua et al., 2024). Pilot trials and observational reports have suggested potential benefits for reducing viral symptoms and mitigating post-acute sequelae (Ferrer et al., 2025; Valerio-Pascua et al., 2024).

These clinical observations are pharmacologically plausible; intranasal delivery provides rapid absorption (Tmax 0.25–3 h), achieves high local concentrations in the upper airway, and partially bypasses hepatic metabolism. Together, these properties align with the therapeutic goals for early viral respiratory illness—namely, reducing viral load at the primary site of replication while limiting systemic adverse effects. Thus, the emerging COVID-19 literature must be interpreted in the context of the route-dependent pharmacokinetics described throughout this review.

This antihistamine is typically administered orally; however, intravenous, intramuscular, and subcutaneous routes have also been documented (Rumore, 1984; Rizvi et al., 2024). Furthermore, the compound’s safety profile, accessibility, and documented efficacy position it as a candidate for modernized delivery platforms, including intranasal sprays and mucoadhesive systems (Rumore, 1984; Rizvi et al., 2024). Interestingly, intranasal routes of this drug have recently been explored (Kandimalla and Donovan, 2005; Huang and Chiou, 1981; Toor et al., 2001).

Despite the renewed clinical interest, published data on CPM’s pharmacokinetics remain scattered across species, formulations, and decades of literature. Prior reviews have summarized antihistamine pharmacology broadly, but none have synthesized CPM’s route-specific and species-specific PK with modern translational applications. A consolidated evaluation is therefore essential to contextualize current findings and inform future development.

This report aims to document the pharmacokinetic characteristics of CPM and assess the bioequivalency and viability of intranasal administration of this drug. To this end, a review approach was carried out to review and analyze the existing literature. Accordingly, this review integrates interspecies comparisons, mechanistic findings, and formulation-dependent variability to clarify how CPM’s pharmacokinetics shape its current and emerging clinical applications.

Despite decades of clinical use, the pharmacokinetic profile of CPM remains unusually complex, with marked variability across species, wide differences in absorption between formulations, and substantial first-pass metabolism shaping systemic exposure. These features make CPM an ideal candidate for revisiting route-dependent pharmacology, particularly intranasal and buccal administration, where absorption kinetics and tissue distribution diverge meaningfully from conventional oral dosing. Understanding these differences is essential not only for optimizing dose selection but also for evaluating emerging therapeutic applications.

## 2 Materials and methods

### 2.1 Protocol and registration

This review followed the Preferred Reporting Items for Systematic reviews and Meta-Analyses extension for Scoping Reviews (PRISMA-ScR) guidelines (Tricco et al., 2018). A scoping review framework was selected because pharmacokinetic studies of CPM span heterogeneous designs, species, formulations, and outcomes, making formal meta-analysis inappropriate. This approach allows mapping the breadth of available evidence while identifying mechanistic and translational themes relevant to modern intranasal and buccal formulations.

The review protocol was not registered in a public database.

### 2.2 Eligibility criteria

Studies were eligible if they:

- Population/Species: Reported pharmacokinetics, bioavailability, or bioequivalence of CPM in humans or non-human animal models.
- Intervention: Administered CPM through any route (oral, intravenous, intranasal, buccal, or other).
- Outcomes: Reported at least one pharmacokinetic parameter (e.g., Cmax, Tmax, AUC, t_1/2), comparative bioavailability data, or mechanistic data relevant to absorption, distribution, metabolism, or excretion.
- Study Design: Included original articles, clinical trials, crossover pharmacokinetic studies, mechanistic transport studies, or comparative formulation studies; both stereospecific and non-stereospecific assessments were included.

This broad eligibility framework ensured that both classical pharmacology studies and recently published intranasal or stereospecific investigations were captured, allowing comparison across species and formulations.

Publication Characteristics: Published in peer-reviewed journals, in English, with full text available.

Studies were excluded if they focused exclusively on histamine antagonists other than CPM, reported *in vitro* data without any *in vivo* pharmacokinetic component, were conference abstracts, editorials, or narrative reviews without original data.

The primary database searched was PubMed/MEDLINE. No additional databases were queried. Reference lists of included studies were screened manually for additional eligible articles.

### 2.3 Search strategy

Three PubMed search queries were performed without date restrictions, most recently updated on 17 August 2025:

- Search 1: “Chlorpheniramine” AND “Bioavailability” (n = 38 records)
- Search 2: “Chlorpheniramine” AND “Bioequivalency” (n = 14 records)
- Search 3: “Intranasal” AND “Chlorpheniramine” (n = 54 records)

All retrieved records were imported into a reference manager, and duplicates were removed. Two reviewers independently screened titles and abstracts for relevance. Full texts of potentially eligible articles were then reviewed against the eligibility criteria. Disagreements were resolved through discussion.

### 2.4 Date charting process

A standardized data extraction sheet was developed. For each included study, the following information was charted:

- Author(s), year, and country
- Study design and setting
- Species (human or specific animal model)
- Sample size and demographics
- Route of administration and formulation
- Dose and dosing regimen
- Pharmacokinetic parameters (Cmax, Tmax, AUC, t_1/2, bioavailability %)
- Comparator formulation or route
- Key mechanistic findings (e.g., mucosal transport, tissue distribution)

**FIGURE 1:**
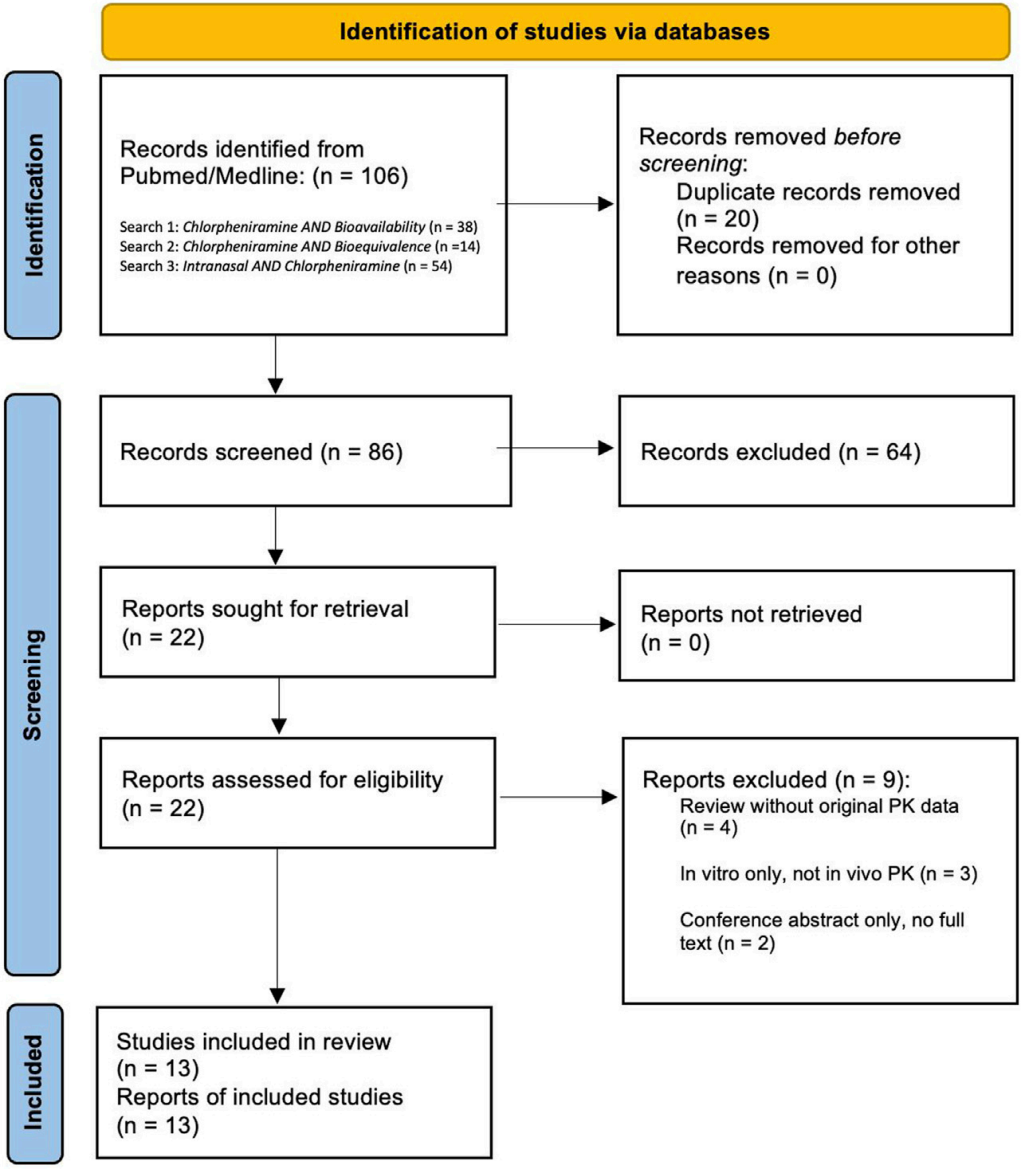
PRISMA flow diagram illustrating the identification, screening, eligibility, and inclusion of studies in the review.

Data extraction (shown in Figure 1) was performed by one reviewer and verified by a second for accuracy. Discrepancies during extraction were resolved through discussion, with particular attention to harmonizing pharmacokinetic parameters reported under differing assay conditions or species-specific models.

### 2.5 Synthesis of results

Pharmacokinetic data in humans were extracted from controlled studies evaluating oral immediate-release and controlled-release formulations (Toor et al., 2001; Kotzan et al., 1982; Vallner et al., 1982; Huang et al., 1982), intravenous administration (Huang et al., 1982), buccal mucoadhesive delivery (Sekhar et al., 2008), and intranasal sprays (Toor et al., 2001). Animal studies included early pharmacology reports in rodents (Huang and Chiou, 1981), stereoselective PK in rats (Chou and Donovan, 1997), bovine studies (Kandimalla and Donovan, 2005), and intranasal administration in dogs (Ichimura and Jackson, 1985; Lung and Wang, 1987).

Clinical outcome data for intranasal CPM in viral respiratory infections, including COVID-19, were derived from randomized controlled trials and observational studies (Rizvi et al., 2024; Torres et al., 2021; Ferrer et al., 2025; Sanchez-Gonzalez et al., 2021; Sanchez-Gonzalez et al., 2022).

Pharmacokinetic data in humans were extracted and synthesized thematically, beginning with oral, intravenous, buccal, and intranasal routes. Comparative bioavailability studies were described separately from mechanistic or distribution studies. No quantitative meta-analysis was performed due to methodological heterogeneity across studies. Narrative synthesis was chosen because methodological heterogeneity, including different sampling schedules, PK modeling approaches, stereospecific analyses, and non-comparable dosing regimens, precluded quantitative pooling. Instead, emphasis was placed on identifying cross-species patterns, formulation-dependent differences, and mechanistic insights.

## 3 Pharmacokinetics of CPM in humans

Human pharmacokinetic data for CPM have been reported across four main administration routes, oral, intravenous, intranasal, and buccal each providing complementary insight into absorption, exposure, and disposition. Oral dosing offers the clearest understanding of systemic kinetics and first-pass metabolism, IV administration defines the intrinsic disposition parameters, while intranasal and buccal routes illustrate the impact of mucosal delivery on onset and bioavailability.

### 3.1 Oral administration

Following oral administration, CPM is rapidly absorbed, reaching peak plasma concentrations (Cmax) within 2–4 h (Tmax) (Rizvi et al., 2024; Huang and Chiou, 1981). The elimination half-life in adults has been reported between 13 and 25 h, with some variability depending on renal function and age (Rizvi et al., 2024). Oral bioavailability is considered moderate (25%–50%) due to substantial first-pass hepatic metabolism (Rizvi et al., 2024; Kandimalla and Donovan, 2005). Multiple studies, including those by Vallner et al. and Kotzan et al., have shown that formulation type—such as syrup, conventional tablets, or controlled-release capsules—can significantly influence systemic exposure (Toor et al., 2001; Kotzan et al., 1982; Vallner et al., 1982).

Food delays the absorption of CPM but does not reduce its extent (Rizvi et al., 2024). In pediatric and geriatric patients, pharmacokinetic parameters vary due to differences in hepatic enzyme expression and renal clearance capacity (Rizvi et al., 2024). New oral dosage forms, including jelly-based systems and bioadhesive films, are being explored to enhance compliance and provide consistent absorption profiles, particularly in populations with dysphagia or altered gastrointestinal physiology (Kim et al., 2020).

Taken together, the oral data highlight the significant influence of first-pass metabolism, gastric emptying, and formulation design on systemic exposure, establishing a baseline for comparison with non-oral routes that partially bypass these limitations.

### 3.2 Intravenous and intramuscular routes

IV administration of CPM provides complete systemic bioavailability and bypasses gastrointestinal and hepatic first-pass effects (Rizvi et al., 2024; Huang and Chiou, 1981). Huang et al. (1982) reported a steady-state volume of distribution of 7–10 L/kg and a half-life of up to 28 h following intravenous injection (Huang and Chiou, 1981). Rumore (1984) similarly noted systemic clearance patterns across different parenteral dosing regimens, making the IV route a valuable reference for comparing alternative delivery systems (Rumore, 1984; Huang and Chiou, 1981).

IM injections, while less frequently used, provide a depot effect with slower absorption and longer duration of action. However, both routes are typically limited to inpatient or emergency use due to the complexity of administration. In clinical scenarios requiring rapid onset, such as anaphylaxis, IV CPM remains a standard tool.

While parenteral routes clarify CPM’s intrinsic disposition and serve as a reference point for systemic exposure, they offer limited insight into mucosal delivery. This makes intranasal administration particularly important, given its potential to alter both the rate and extent of absorption through highly vascularized tissues.

### 3.3 Intranasal administration in humans

Intranasal Chlorpheniramine (iCPM) has gained attention due to its non-invasive nature, rapid systemic absorption, and potential for direct central nervous system access. A pharmacokinetic study by Van Toor et al. demonstrated that plasma levels after intranasal administration were comparable to oral dosing, with Tmax values between 0.25 and 3 h (Toor et al., 2001). Clinical data from Sanchez-Gonzalez et al. and Torres et al. have further confirmed the effectiveness of iCPM in treating allergic rhinitis and COVID-19, showing symptom relief and avoidance of hospitalization in acute cases due to its demonstrated inhibition of viral adsorption, viral replication reduction, and virucidal effect against SARS-CoV-2 (Torres et al., 2021; Sanchez-Gonzalez et al., 2021).

Likewise, Ferrer et al. demonstrated that iCPM also offers significant potential for managing both acute COVID-19 and long COVID by integrating H1 receptor antagonism and T2R activation (Ferrer et al., 2025). This dual-target mechanism demonstrated histamine-mediated suppression of inflammation, which mitigates cytokine storms and enhances mucosal immunity through the production of nitric oxide, synthesis of antimicrobial peptides, and mucociliary clearance (Ferrer et al., 2025). Targeting the upper respiratory tract, iCPM reduces viral replication while minimizing systemic side effects, making it a versatile and accessible treatment option. A study from Valerio-Pascua et al. provides strong evidence that iCPM significantly reduces the incidence of Post-Acute Sequelae Complications (PASC) symptoms (Valerio-Pascua et al., 2024).

These findings, aligned with prior pharmacokinetic and clinical evidence, underscore the potential of iCPM as a versatile therapeutic strategy, capable of combining rapid systemic absorption with targeted modulation of mucosal immunity and inflammation. Nevertheless, the nasal route faces challenges, including mucociliary clearance and active efflux via P-gp and MRP1 transporters. Kandimalla and Donovan demonstrated that CPM is actively transported out of the nasal mucosa, limiting its brain penetration (Kandimalla and Donovan, 2005). New strategies, such as co-formulating with permeation enhancers or using bioadhesive gels, are being investigated to overcome these limitations (Rizvi et al., 2024; Sekhar et al., 2008).

Overall, the intranasal route demonstrates rapid absorption, early peak concentrations, and partial avoidance of first-pass metabolism features that distinguish it markedly from oral administration. These kinetic advantages, combined with evidence of transporter-mediated limitations and local mucosal effects, provide a mechanistic foundation for the clinical observations discussed later, including studies in allergic rhinitis and viral respiratory disease.

### 3.4 Buccal administration

In contrast to the rapid nasal absorption, buccal delivery provides a slower but more controlled mucosal uptake, representing another non-invasive route that bypasses part of the hepatic first-pass effect. The buccal route allows partial bypass of first-pass metabolism, offering consistent systemic absorption. Sekhar et al. developed a hydroxyethyl cellulose-based mucoadhesive patch delivering CPM at a rate of 0.14 ± 0.03 mg h^-1^·cm^-2^, achieving higher bioavailability than standard oral tablets (Sekhar et al., 2008). Approximately 45% of the drug was absorbed within 16 min, with a rapid onset noted in human volunteers.

Despite favorable kinetics, buccal delivery systems have not yet seen widespread clinical implementation, in part due to unfamiliarity among patients and regulatory hurdles. However, their potential for use in pediatric and geriatric populations, or as a platform for dual-drug delivery (e.g., CPM with corticosteroids), warrants further investigation (Rizvi et al., 2024). These characteristics make buccal administration a useful comparator for understanding how different mucosal surfaces influence CPM absorption kinetics and support its development for populations where oral or intranasal dosing may be impractical.

To contextualize the human pharmacokinetic profile, it is necessary to examine how CPM behaves across preclinical species, particularly regarding half-life, tissue distribution, and transporter interactions. These interspecies data provide mechanistic insight and clarify which findings translate most reliably to human dosing.

## 4 Pharmacokinetics of CPM in animal models

Animal models provide essential mechanistic context for CPM pharmacokinetics, highlighting how differences in protein binding, hepatic metabolism, mucosal transport, and vascular physiology influence systemic exposure. Rodent, rabbit, canine, bovine, and equine studies collectively illustrate the degree to which species-specific physiology shapes absorption and clearance, thereby informing the translational relevance of non-human findings to human dosing.

### 4.1 Rodents and rabbits

**FIGURE 2:**
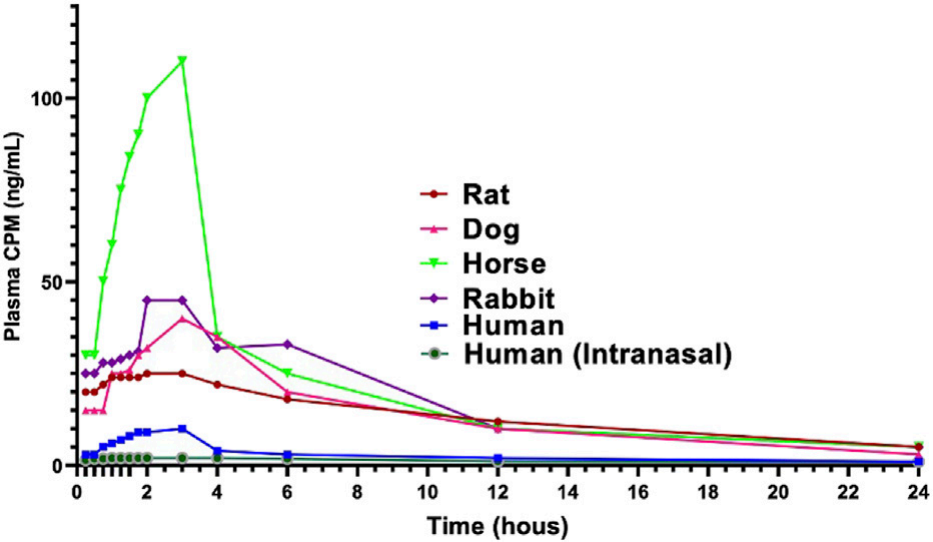
Comparative model of plasma chlorpheniaremine maleate (CPM) pharmacokinetics across species following intravenous (IV) or intranasal (IN) administration. Data were compiled from previously published studies in rats, rabbits, horses, and humans (Rumore, 1984; Kandimalla and Donovan, 2005; Ichimura and Jackson, 1985; Lung and Wang, 1987; Huang and Chiou, 1981; Monaikul et al., 2023) and from the Experimur Beagle-dog intranasal PK study (2023). The figure was created by the authors to illustrate interspecies differences in plasma concentration–time profiles.

Rodents and rabbits offer the clearest insight into CPM’s basic disposition mechanisms, including tissue distribution, hepatic extraction, and protein binding dynamics. Rodents and rabbits have served as essential models to characterize the pharmacokinetics of CPM. Figure 2 compares single doses between different species. In a landmark study, Huang and Chiou (1981) demonstrated rapid intravenous distribution of CPM in rabbits, with high tissue-to-plasma concentration ratios in lungs (160-fold), kidneys (80-fold), and brain (31-fold) (Huang and Chiou, 1981). The volume of distribution reached 10.8 L/kg, and the hepatic extraction ratio was calculated at 0.88 (Lung and Wang, 1987). This high tissue affinity was attributed in part to relatively low plasma protein binding in rabbits (44%), compared to humans (∼70%).

These species have also been used to characterize demethylated metabolites and simulate dose-dependent kinetics. Oral administration confirmed a substantial presystemic effect. These animal data have helped predict human bioavailability and inform toxicology assessments, especially regarding the CNS effects and accumulation in renal or hepatic dysfunction models (Huang and Chiou, 1981). These characteristics make rodent and rabbit models particularly useful for understanding how physicochemical factors and protein binding drive CPM distribution, findings that partially translate to humans and help explain the drug’s long terminal half-life.

### 4.2 Canine and bovine models

In contrast to rodents and rabbits, canine and bovine models have been especially informative for evaluating mucosal and intranasal delivery of CPM. These species provide direct insight into nasal vascular responses, transporter-mediated efflux, and mucosal permeability, factors that are highly relevant to modern intranasal formulations.

**FIGURE 3:**
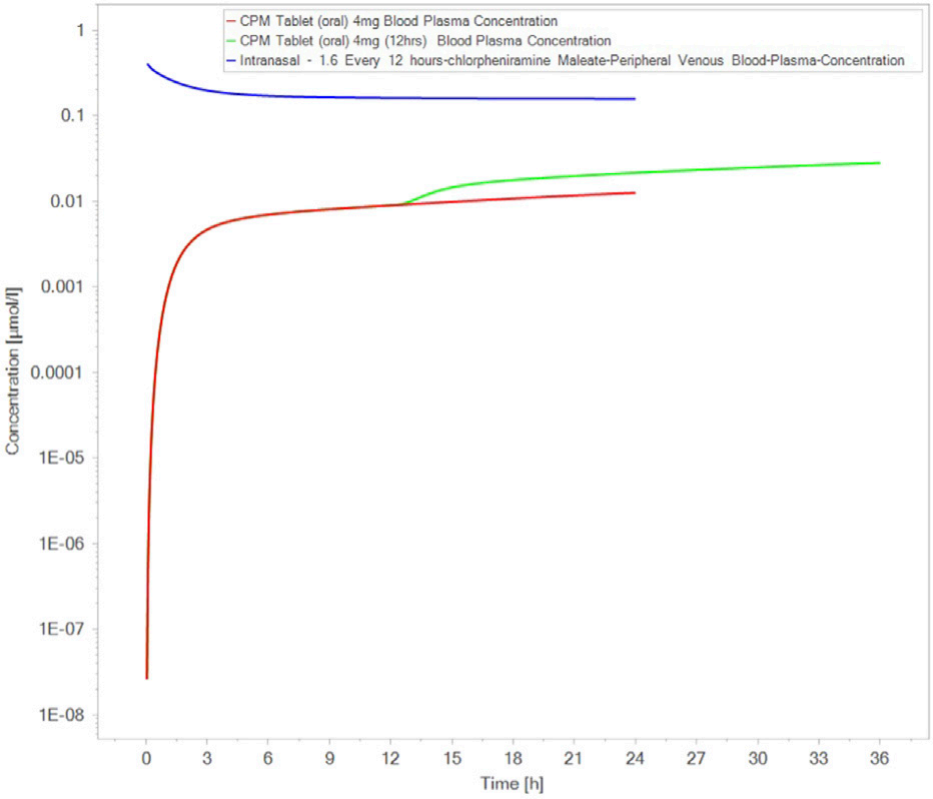
Comparison of intranasal route bioavailability between dogs and humans. Simulated comparison of blood plasma concentrations of chlorpheniaremine maleate (CPM) following oral and intranasal administration. Concentration–time profiles were modeled using published human pharmacokinetic parameters (Toor et al., 2001; Kotzan et al., 1982; Vallner et al., 1982; Huang et al., 1982; Sanchez-Gonzalez et al., 2021; Sanchez-Gonzalez et al., 2022). The figure was generated by the authors and does not reproduce any previously published material.

Canine models, particularly regarding nasal delivery, have shown distinctive responses to CPM (Ichimura and Jackson, 1985). Found that CPM induced intense vasoconstriction in isolated canine nasal blood vessels, which differed significantly from other H1 antihistamines (Ichimura and Jackson, 1985). (Lung and Wang, 1987) reported that CPM caused differential effects on nasal vascular and airway resistance in dogs, suggesting that localized vascular tone could impact drug absorption in intranasal formulations (Lung and Wang, 1987). Figure 3 compares the bioavailability of the intranasal route between dogs and humans (Lung and Wang, 1987).

More recent data further confirm the rapid intranasal absorption of CPM in canines. In the non-GLP single-dose study (Monaikul et al., 2023), two female Beagle dogs received 100 µL of a 70 mg/mL chlorpheniramine maleate solution per nostril (200 µL total). Peak plasma concentrations were observed at 5 min (T_max = 0.083 h) with mean C_max ≈ 472 ng/mL (range 295–650 ng/mL) and AUC_0_–_8h_ ≈ 345 ng h/mL. Plasma levels declined rapidly to ≈1% of C_max by 8 h, indicating fast mucosal uptake and short systemic persistence in this model. No treatment-related clinical signs were reported. These findings corroborate earlier canine vascular studies and quantitatively demonstrate that the intranasal route achieves very rapid systemic exposure while remaining well-tolerated. Together, they strengthen the translational rationale for nasal CPM formulations in humans.

Taken together, canine data emphasize that CPM’s intranasal absorption is rapid, concentration-dependent, and heavily influenced by local vascular and transporter activity. These mechanistic features observed in canine and bovine nasal studies rapid mucosal uptake, vascular responsiveness, and transporter-mediated efflux closely parallel those documented in human intranasal studies, reinforcing their translational relevance.

### 4.3 Equine models (Thoroughbred horses)

Equine pharmacokinetic studies extend the interspecies comparison by illustrating how metabolic rate and clearance can drastically shorten CPM’s half-life in large mammals. In equine pharmacology (Kuroda et al., 2013), evaluated d-chlorpheniramine in Thoroughbred horses using both intravenous and oral routes (Kuroda et al., 2013). Oral bioavailability was low (approximately 38%), with peak concentrations achieved at 1 h and pharmacodynamic response evident at 2 h post-dose (Kuroda et al., 2013).

The IV formulation showed rapid clearance and a short half-life (∼2.7 h), further reinforcing interspecies differences. The low oral bioavailability suggests that substantially higher doses may be needed in equine medicine compared to other species. These findings emphasize the importance of species-specific pharmacokinetics when translating dosing regimens from preclinical models to veterinary or human settings (Kuroda et al., 2013). This rapid clearance contrasts sharply with the prolonged half-life in humans, reinforcing that extrapolation across species must account for differences in hepatic metabolism, body size, and protein binding capacity.

Together, the animal data illustrate how species-specific differences in clearance, protein binding, and mucosal absorption shape pharmacokinetic behavior. These findings provide a mechanistic backdrop for understanding why certain CPM formulations perform inconsistently across studies. Building on these insights, the following section evaluates how formulation design influences CPM bioavailability in both human and preclinical systems.

## 5 Comparative bioavailability and bioequivalence studies

Beyond species differences, formulation-dependent behavior markedly influences CPM exposure. Understanding the interplay between dosage form and pharmacokinetics is essential when comparing data across routes and species. The impact of formulation on CPM’s pharmacokinetics has been well-documented (Kotzan et al., 1982). and (Vallner et al., 1982) demonstrated that controlled-release formulations extended the time to peak concentration but did not yield bioequivalence in AUC compared to conventional forms. In fact, controlled-release products often produced incomplete absorption, and their AUC values were not dose-proportional to the regular-release counterparts (Kotzan et al., 1982; Huang et al., 1982).

Bui et al. (2000) added another layer by studying the stereospecific bioavailability of racemic CPM. They observed significant differences in systemic exposure between enantiomers, with (+)-CPM showing higher Cmax and AUC compared to the (−) enantiomer (Hiep et al., 2000). These findings indicate that both formulation and stereochemistry affect CPM’s pharmacokinetics. Recent comparisons between jellies, syrups, and tablets in dogs confirmed that jellies and syrups had comparable absorption. In contrast, tablets delayed absorption and reduced the maximum concentration (Cmax), possibly due to prolonged disintegration and pH-dependent solubility (Kim et al., 2020). These findings reinforce the regulatory and biopharmaceutical challenges in developing consistent and interchangeable dosage forms. Table 1 summarizes the reviewed studies.

**TABLE 1:** Summary of pharmacokinetic, bioavailability, and mechanistic studies of chlorpheniramine maleate across species.

Author	Species/Population	Study design	Administration route	Treatment/Intervention	Findings
Lung and Wang (1987)	Human n = 12	Bioequivalence study (stereospecific vs. nonspecific formulations)	Oral	Racemic CPM. Treatments: two different 8 mg of racemic Chlorpheniramine and 75 mg of phenylpropanolamine formulas	Tmax = 8.7 h, Cmax = 6.8–20.2 ng/mL Stereospecific enantiomers (−) are less bioequivalent than those (+) Cmax and AUC values were higher for (+)S-chlorpheniramine ((+)S-CPAM) compared to (−)R-chlorpheniramine ((−)R-CPAM) (13.3 vs. 6.8 ng/mL and 409 vs. 222 ng/ml/h, respectively) while Clt/F and Vd/F were lower (9.8 vs. 17.61/h and 321 vs. 627 1, respectively). No difference was observed for Tmax and t½
Chou and Donovan (1997)	Rat n = 10	Mechanistic study (*in vitro*, rat nasal mucosa, transport/efflux)	I.V., Intranasal	The nasal cavity was isolated from the respiratory and gastrointestinal tracts using a modification of the method of Hussain et al. 100 μL drug solution IV for nasal administration, 50 µL drug solution	There were no measurable concentrations of CPM in the CSF following either intraarterial or intranasal administration—no bioavailability of intranasal CPM in the CSF.
Huang et al. (1982)	Human n = 5	PK study (human, IV vs. oral)	I.V., Oral	Single oral (10 mg./10 mL diluted in water), 2 4 mg tablets or IV (5 mg) dose was administered collecting samples at 2.5, 5, 10, 20, 30, 45, 60, 90 min and 2, 4, 6, 12, 24, 36 and 48 h after dosing (IV) at 30, 45, 60, 90 min and 2, 4, 6, 8, 10, 12, 24, 36 and 48 h after dosing	The average peak time after single oral dosing was 2.8 h with an average peak level of 17.9 ng/mL half-lives obtained in this study (20.6–43.4 h)
Huang and Chiou (1981)	Rabbit n = 6	PK study (rabbit, I.V., tissue distribution)	I.V	CPM Injection 10 mg/ml or 7.03 mg/mL Bolus of 3 mg/kg Plasma collection usually at 2, 10, 20, 40, 60 min, and 2, 3, 4, 6, 8, 10, 12, and sometimes 24 h after injection	The harmonic mean of the half-life in six rabbits. (A-F) was 2.57 h with a range of L 72–4.87 h. These values are similar to those (mean 1.7 h). Found in dogs, but were much shorter than reported for humans 28 h from Peets et al., 115.6 h from Thompson and Leffert, and 22.5 h from Huang et al—most extensive distribution in lungs, Brain, and Spleen. The lower percentage of serum protein binding in rabbits, as compared to those of dogs and humans (44% vs. 70% and 73%), might have contributed in part to the larger volume of distribution in rabbits (Varea of 15.5 L/kg vs. 5.25 and 3.36 L/kg)
Ichimura and Jackson (1985)	Dog N = 6	Mechanistic study (dog, nasal vascular responses)	N/A	CPM titration in nasal mucosal smooth muscle vascular beds	Chlorpheniramine maleate and pyrilamine maleate caused the maximal contraction at a dose of 6 × 10 · 3 M. Chlorpheniramine induced the most significant absolute response. CPM antihistamine does not need extracellular calcium ions to induce contraction in this tissue. Histamine does need extracellular calcium ions to cause contraction. Antihistamines may evoke direct contraction of blood vessels by depolarizing smooth muscle cell membranes coupled with a release of internally bound calcium ions
Kandimalla and Donovan (2005)	Cow N = not specified	Mechanistic Study (*in vitro*, bovine nasal mucosa, transporter assay)	N/A	Sweetana-Grass was used to determine the permeability of Chlorpheniramine and chlorcyclizine through the bovine olfactory mucosa	The submucosal to mucosal fluxes (Js–m) of CPM across the olfactory mucosa were significantly greater than the mucosal to submucosal fluxes (Jm–s). Moreover, the submucosal–mucosal permeability of CPM was temperature dependent and saturable. These results indicate that CPM is effluxed from the olfactory mucosa by efflux transporters such as P-gp and MRP1. Transport studies across inert polymeric membranes demonstrated that the permeability of CPM decreased at donor concentrations higher than 3 mM, suggesting that physicochemical properties such as self-aggregation also play a role in the reduced olfactory mucosal permeability of these compounds at higher concentrations
Kim et al. (2020)	Dog N = 15	Bioavailability study (dog, oral, jelly vs. tablet formulation)	Oral	CPM jelly 1.875 mg/15 gr. of jelly Acetaminophen and dextromethorphan were mixed	The absorption of CPM was more delayed and decreased by Tablet-H than by AAP. According to several reports, CPM’s absorption rate is slow, probably because the absorption of the weakly basic drug, CPM, occurs mainly in the small intestine and is hardly absorbed in the stomach. According to the FDA’s criteria, the syrup, jellies, and tablets were not bioequivalent. Even so, the jellies and syrup showed similar absorption rates and extents. On the other hand, the tablets significantly delayed and reduced the absorption of the cold medicines compared to the syrup
Kotzan et al. (1982)	Human n = 15	Bioavailability study (human, regular vs. controlled-release)	Oral		The AUC of the controlled release formu-lations was not equivalent to the exact amounts of non-controlled release products, nor were they equal to two times the A UC of the 4-mg syrup, realizing the difficulties involved in obtaining an exact A UC. The controlled-release products also extended the time necessary to attain peak drug levels compared to the 4-and 8-mg
Kuroda et al. (2013)	Horses n = 4	PK/PD study (horse, IV and oral)	I.V., Oral	The IV dosing study was conducted using two dose rates (0.1 and 0.5 mg/kg BW) of CPM solution For oral 0.5 mg/kg as well. Diluted the ion in 500 mL of water	The parameters estimated from the pharmacokinetic/pharmacodynamic link model, Emax, EC50, and h, were 79.2% ± 12.1%, 53.2 ± 16.1 ng/mL, and 0.88 ± 0.42, respectively. The effect of 0.1 mg/kg IV and 0.5 mg/kg P.O. doses was very low; therefore, they were not applied to the pharmacokinetic/pharmacodynamic link model. Very poor bioavailability of oral CPM in Horises’ low bioavailability (approximately 38%) indicates that a P.O. dose of 2.6 times the IV dose may be necessary
Monaikul et al. (2023)	Beagle dog (n = 2, female)	Non-GLP pharmacokinetic study	Intranasal	Single administration of chlorpheniramine maleate (CPM) 70 mg/mL solution (100 µL per nostril; 200 µL total dose)	After intranasal dosing, rapid absorption was observed with Tmax = 5 min (0.083 h) and mean Cmax = 472.5 ng/mL (range 295–650). Mean AUC_0_–_8_ = 344.5 ng h/mL. Plasma concentrations declined to ∼1% of Cmax by 8 h, indicating rapid clearance. No clinical adverse signs were reported. Study supports fast systemic exposure and good tolerability following nasal administration
Lung and Wang (1987)	Dog n = 9	Mechanistic study (dog, nasal airway resistance)	Intranasal	Dogs received intranasal Chlorpheniramine (CPM) at doses <1 mg and >1 mg, while nasal airway resistance and vascular resistance were continuously measured under humidified airflow	At doses of <1 mg, CPM increases vascular resistance and decreases airway resistance. A dose>1 mg, CPM decreases vascular resistance. In doses of less than 1 mg, increased nasal vascular resistance but decreased nasal airway resistance, suggesting a constrictor action on the arterioles and a decrease in vascular capacitance by opening arteriovenous anastomoses or dilatation of the muscular venules. However, when doses higher than 1 mg were given, nasal vascular resistance decreased significantly, while nasal airway resistance decreased only slightly. This suggests that the drug in high doses causes dilatation of the arterioles but has a negligible effect on the capacitance vessels
Sekhar et al. (2008)	Human n = 8	PK/Buccal delivery study (human, mucoadhesive buccal patches)	Oral, Buccal	Oral/buccal intervention: Volunteers received a phosphate buffer solution (20 mL, pH 6.6) containing 4 mg of chlorpheniramine maleate (CPM) and phenol red (20 μg/mL). They were instructed to swirl the solution in the mouth (∼60 times/min). Sequential oral cavity samples were collected at fixed intervals, and after completion, the remaining solution plus two buffer rinses were pooled. Drug content was quantified using HPLC.	The results of the buccal absorption study (Figure 2) revealed that CPM could be absorbed through the oral mucosal membranes. We found that ∼45.90% of the drug was absorbed in 16 min. The drug was absorbed at a rapid rate for the first 4 min, after which the drug absorption continued at a uniform rate. The volunteers did not swallow the solution. This was evident from the observation that the total quantity of phenol red (392.40 ± 5.31 μg)
Vallner et al. (1982)	Human n = 15	PK/PD study (human, multiple oral dosing)	Oral	Blood samples were collected before drug administration on day 3, day 6, and day 7 (the study day) at Oh (i.e., before the 6 a.m. dose). Then at 1, 2, 3, 4, 6, 7, 8, 9, 12, 24, 36, 48, 72, 96, and 144 h after that	The disappearance curves of the three products are essentially parallel, indicating similar half-lives. The half-lives calculated from these curves are 24·5 h for the repeat action tablets, 25·4 h for the barrier coated-bead capsules, and 25· 1 h for the conventional release tablets
(Toor et al., 2001)	Human n = 15	PK/Bioavailability study (human, intranasal vs. oral)	Oral, Intranasal	The blood samples for plasma drug levels were taken before, and at 0.25, 0.50, 1.0, 2.0, 3.0, 4.0, 6.0, 8.0, 12, 16, and 24 h after drug administration. Additional blood samples were obtained at 36, 48, and 72 h after oral administration of CPM only	Nasally applied chlorphenamine maleate was readily absorbed, reaching peak plasma levels mainly between 0.25 and 3.0 h after ap-plication. The initial steep increase in plasma drug levels was followed by a gradual decrease over time up to 24 h after dosing, and was associated with secondary and tertiary peaks

These clinical findings align with the pharmacokinetic properties of intranasal CPM, particularly its rapid absorption (Tmax 0.25–3 h), high early local concentrations, and partial first-pass bypass. These PK attributes are consistent with the therapeutic goals for early-stage viral infections, where rapid mucosal action and dense nasopharyngeal exposure may meaningfully influence viral replication dynamics.

## 6 Discussion

This discussion integrates human and animal pharmacokinetic findings, formulation-dependent variability, and mechanistic insights to highlight how CPM’s absorption, distribution, and clearance patterns shape its clinical applications. By synthesizing route-specific characteristics with interspecies data, this section contextualizes CPM’s therapeutic potential particularly for intranasal and transmucosal delivery and identifies the translational implications for modern clinical use.

Across studies, CPM demonstrated highly variable pharmacokinetics depending on species, formulation, and route of administration (Table 2). In humans, oral immediate-release formulations showed a Cmax of 5.7 ng/mL at 2.2 h after a single 4 mg dose, with an AUC of 57.9 ng h/mL (Vallner et al., 1982). Controlled-release and multiple-dose studies reached steady-state Cmax of 25.9–32.5 ng/mL with extended Tmax of 3.9–8.4 h and AUC 837–1,202 ng h/mL (Hiep et al., 2000; Kotzan et al., 1982; Vallner et al., 1982; Huang et al., 1982). Buccal mucoadhesive delivery (4 mg) achieved Cmax of 6.2 ng/mL at 3.3 h with AUC 85.0 ng h/mL (Sekhar et al., 2008). Intranasal CPM (0.4%) in healthy males reached peak plasma concentrations between 0.25 and 3.0 h (Toor et al., 2001). Intravenous bolus dosing (5 mg) yielded a terminal half-life of 22–23 h (Huang et al., 1982). In practical terms, these parameters indicate that CPM exposure is highly dependent on both formulation and absorption site, which must be considered when interpreting clinical effectiveness across delivery routes.

**TABLE 2:** Interspecies comparison of pharmacokinetic parameters of Chlorpheniramine in different administration routes.

Species	Route	Cmax (ng/mL)	Tmax (h)	AUC (ng-h/mL) or F (%)	T^1/2^ (h)	Key notes
Human	Oral (IR tablet,4 mg)	5.73 ± 1.08	2.17 ± 0.41	57.85 ± 15.50	NR	Single-dose, healthy volunteers
	Oral (steady-state; conventional & CR products)	25.9–32.5	3.9–8.4	837–1,202	24.5–25.4	Multiple-dose, day-7 steady-state across products; ranges extracted from tables
	Intranasal (0.4%)	NR	0.25–3.0	NR	NR	Healthy males (n = 24); peak window reported
	Buccal (mucoadhesive patch, 4 mg)	6.16 ± 0.99	3.33 ± 0.82	84.99 ± 17.96	NR	Single-dose, healthy volunteers
	Intravenous (5 mg bolus)	N/A	0 (IV)	NR	22–23	Two subjects; terminal half-lives reported in summary
Horse	Oral (0.5 mg/kg d-CPM)	65.0 ± 12.3	1.20 ± 0.45	273 ± 82.8	2.12 ± 0.71	n = 5; d-isomer only; one-compartment PO model
Rabbits	Intravenous (0.5–3 mg/kg)	N/A	0 (IV)	NR	2.57 (range 1.72–4.87)	Harmonic mean t½ across six NZW rabbits
Dog	Intranasal (single dose, 0.4% CPM spray)	472.5 ± 250 (range 295–650)	0.083 (5 min)	344.5 ± 73.0	∼1.6–1.7	Two female Beagle dogs received a single 200 µL total dose (100 µL per nostril) of 70 mg/mL chlorpheniramine maleate solution. Rapid absorption observed with peak levels at 5 min and >99% decline by 8 h. No adverse events reported
	Intravenous	NR	NR	NR	1.7 h (elimination)	Distribution t½α 12.5 min; Vdβ 525% BW; F_Oral: up to 39.4% depending on dose
	Oral (50–200 mg)	NR	NR	F = 9.4–39.4%	NR	Absolute bioavailability varied with dose (50–200 mg solutions)
Rat	Intranasal	NR	NR	NR	NR	Tissue distribution studies only; no systemic Cmax, Tmax, or AUC reported
	Intravenous (Racemate)	NR	NR	Higher AUC for (+)-S enantiomer	18.2 min ((−)); 50 min (+)	Stereoselective PK after IV; clearance differences and protein binding noted
	Oral/IV (20 mg/kg racemate)	NR	NR	Higher AUC for (+)-S enantiomer	NR	Stereoselective absorption/metabolism in rats
Cow	*Ex vivo* nasal	NR	NR	NR	NR	Distribution studies in bovine nasal mucosa: drug retention but no systemic PK reported

Taken together, these data underline three consistent pharmacokinetic themes: (i) CPM displays rapid absorption with marked formulation dependence; (ii) a long elimination half-life supports less frequent dosing than traditionally prescribed; and (iii) extensive tissue distribution and high protein binding prolong systemic exposure. These features provide a mechanistic basis for the variability observed across studies and guide the interpretation of intranasal and buccal delivery, where partial avoidance of first-pass metabolism and high mucosal vascularity alter both onset and exposure.

With a serum half-life (t½) of ∼20 h in adults, CPM’s elimination from the body is primarily by metabolism to monodesmethyl and desmethyl compounds. The t½ is increased in the presence of renal dysfunction, whereas it is decreased in children (Rumore, 1984). The exact mechanism of presystemic first-pass elimination and the effects of dose levels on this process are currently unclear. It has been reported that CPM is predominantly metabolized by cytochrome P450 2D6 (CYP2D6) (Huang et al., 1982). Biopharmaceutical and pharmacokinetic studies in humans after single or multiple doses reveal wide interindividual pharmacokinetic variations.

The usual recommended oral dose for adults is 4 mg q4-6 h, or 8–12 mg in the form of sustained-release tablets two to three times daily. The recommended oral dose for children aged six to eleven is 2 mg q4-6 h and 1 mg q4-6 h for children aged two to five. Intravenous, intramuscular, or subcutaneous injection doses of 5–40 mg have also been reported (Rumore, 1984). Understanding CPM disposition in animal models is essential for interpreting its mechanistic behavior and translational applicability. Species-specific differences in metabolism, protein binding, mucosal transport, and vascular physiology provide a foundation for explaining why certain routes, especially intranasal delivery, behave differently in humans than in preclinical systems.

Animal studies revealed distinct pharmacokinetics. Interspecies differences are mostly related to the ability to metabolize the drug, which seems to be elevated in horses (Kuroda et al., 2013). In horses, oral administration of d-CPM (0.5 mg/kg) produced a Cmax of 65.0 ng/mL at 1.2 h with an AUC of 273 ng h/mL and a t½ of 2.1 h (Kuroda et al., 2013). In rabbits, IV administration yielded a harmonic mean half-life of 2.6 h (Huang and Chiou, 1981). Rats exhibited stereoselective PK, with the (+)-S enantiomer showing prolonged half-life (50 min) compared with the (−)-R enantiomer (18.2 min) (Sakurai et al., 1992; Tung et al., 2001).

In dogs, intranasal delivery significantly altered nasal vascular resistance and blood flow, consistent with rapid systemic absorption (Ichimura and Jackson, 1985; Lung and Wang, 1987). In a bovine model, the intranasal route exhibits dose- and pH-dependent limitations, as saturation dynamics (maximal permeability of 15–25 cm/s) impede permeability at drug concentrations exceeding 3 nM and an optimal pH of 6.2 (Kandimalla and Donovan, 2005). In cattle, *ex vivo* nasal studies demonstrated measurable distribution and receptor-mediated effects (Kandimalla and Donovan, 2005).

Clinical studies of intranasal CPM in COVID-19 demonstrated efficacy in early symptomatic relief. A randomized controlled trial reported reduced symptom scores and viral clearance compared to placebo (Sanchez-Gonzalez et al., 2021). Observational studies confirmed clinical improvement in larger patient cohorts (Ferrer et al., 2025), and a pooled analysis highlighted potential benefit in reducing post-acute sequelae (“long COVID”) (Valerio-Pascua et al., 2024).

### 6.1 Interspecies variability in pharmacokinetics

The comparative analysis revealed striking differences in half-life across species. Rabbits displayed rapid elimination (t½ ∼2.6 h), while humans consistently showed prolonged persistence (>20 h), in line with earlier reports of strong protein binding and slow clearance. Dogs and horses exhibited intermediate values, underscoring the role of species-specific physiology, including protein binding capacity and hepatic metabolism, in shaping CPM disposition. These differences limit the direct extrapolation of preclinical data to humans but provide mechanistic insight into the determinants of CPM pharmacokinetics.

### 6.2 Mechanistic insights from animal and In vitro studies

Efflux transporters represent a key limitation to CPM’s effectiveness, particularly in nasal and CNS delivery (Kandimalla and Donovan, 2005). Confirmed that CPM is transported out of the nasal mucosa by P-gp and MRP1, reducing its brain availability. This active transport was energy-dependent, temperature-sensitive, and inhibited by verapamil and quinidine, confirming transporter specificity (Kandimalla and Donovan, 2005).

In addition to transporter effects, CPM shows moderate protein binding (∼70%) and wide tissue distribution (Ichimura and Jackson, 1985). Demonstrated that CPM’s action on nasal vasculature might involve direct smooth muscle activation independent of extracellular calcium, suggesting an alternate vasoconstrictive mechanism (Ichimura and Jackson, 1985). Such effects could alter mucosal blood flow and modulate drug absorption dynamics (Ichimura and Jackson, 1985).

These insights underscore the necessity of considering both drug formulation and mucosal physiology in designing nasal and transmucosal systems. Carrier systems or transporter inhibitors may significantly improve CPM bioavailability in future formulations (Rizvi et al., 2024; Kandimalla and Donovan, 2005). Mechanistically, these observations suggest that CPM is governed by a balance between rapid mucosal uptake and active efflux constraints, resulting in high early concentrations but limited sustained nasal retention. This interplay explains the rapid onset seen in intranasal human studies and provides a rationale for novel formulation strategies aimed at enhancing residence time or transiently modulating efflux activity.

### 6.3 Consideration for dosing frequency

Given that Chlorpheniramine in adults exhibited a terminal elimination half-life consistently in the ∼20–25 h range across intravenous and multiple-dose oral studies (Kotzan et al., 1982; Vallner et al., 1982; Huang et al., 1982), a 12-h dosing interval is pharmacokinetically coherent and avoids unnecessary re-dosing at 6-h intervals. In the human PK literature synthesized in our manuscript, the parallel terminal slopes after repeated oral administration yielded half-lives ≈of 25 h, and intravenous studies similarly reported terminal half-lives of ∼22–23 h (Vallner et al., 1982; Huang et al., 1982); intranasal administration achieved rapid absorption (Tmax ≈0.25–3 h) with systemic exposure comparable to oral dosing (Toor et al., 2001; Sanchez-Gonzalez et al., 2021; Sanchez-Gonzalez et al., 2022).

Under these conditions, with k = ln2/t_1/2 ≈ 0.028–0.035 h^-1^, the expected steady-state fluctuation across a 12-h interval remains modest (Cmax, ss/C_min, ss ≈ e^{kτ} ≈ 1.3–1.5), indicating sustained H1-receptor coverage over each dosing interval without requiring q6 h administration. This PK profile, together with the practical objective of day–night symptom control and the rapid onset afforded by the intranasal route, supported the selection of a twice-daily regimen in our challenge-study schedule (Day 0–5), aligning clinical operations with the drug’s long terminal half-life and ensuring adequate exposure over 24-h cycles.

### 6.4 Translational link between PK and clinical applications

The combined pharmacokinetic evidence across species and administration routes clarifies why CPM performs differently in specific clinical scenarios. The long elimination half-life and extensive tissue distribution observed in humans support infrequent dosing and sustained receptor occupancy, whereas the rapid absorption and early peak concentrations following intranasal delivery make this route particularly suited for acute symptom relief and potential antiviral action. These kinetic distinctions provide a mechanistic foundation for matching CPM’s pharmacokinetic properties to therapeutic objectives.

Preclinical data also contextualize clinical observations. Canine and bovine nasal studies demonstrate that mucosal vascularity, local vasoconstriction, and transporter-mediated efflux significantly influence CPM uptake. These mechanistic features align closely with findings from human intranasal studies, which show fast absorption, limited central nervous system penetration, and high early mucosal exposure factors relevant in conditions such as allergic rhinitis and early viral infection.

Taken together, the integration of PK behavior and mechanistic data establishes a coherent translational framework. This synthesis supports ongoing efforts to design optimized intranasal, buccal, and modified-release formulations while informing dose selection, therapeutic timing, and future clinical study designs.

### 6.5 Clinical implications

Human studies have consistently shown the prolonged half-life and systemic persistence of CPM, supporting its efficacy as a long-acting antihistamine. However, variability in absorption highlights the importance of formulation design. Buccal and intranasal administration appear promising for rapid symptom relief in allergic conditions, while oral controlled-release products demonstrated inconsistent systemic exposure. The favorable tolerability profile observed across human studies, coupled with dose-dependent vascular effects in animals, supports the continued clinical use of CPM, while also emphasizing the need for careful dose selection. These findings collectively highlight how CPM’s route-specific pharmacokinetic behavior can be leveraged to match clinical objectives rapid mucosal action for viral or allergic indications versus systemic persistence for chronic inflammatory conditions.

### 6.6 Future perspective and challenges

Despite its long history of clinical use, substantial knowledge gaps remain in the pharmacokinetics of CPM, particularly regarding modern delivery systems. Pharmacokinetic characterization of contemporary formulations such as nasal sprays, bioadhesive gels, and buccal films has largely been limited to small, heterogeneous studies, often with inconsistent endpoints and non-standardized sampling schedules. As a result, the comparative performance of these formulations remains difficult to interpret, and optimal dosing strategies are yet to be established (Rizvi et al., 2024; Torres et al., 2021; Kim et al., 2020).

Another key challenge is the significant interindividual variability observed in CPM exposure. Polymorphisms in CYP2D6, which plays a central role in CPM metabolism, along with age-related physiological changes, renal dysfunction, and comorbid conditions, may profoundly influence drug disposition. These factors warrant systematic evaluation, particularly in populations most likely to benefit from non-oral delivery routes, including pediatric, geriatric, and respiratory-compromised patients (Rizvi et al., 2024; Lung and Wang, 1987).

Several translational questions also require clarification. Although intranasal CPM has shown encouraging results in allergic rhinitis and early COVID-19 treatment, critical parameters such as dose–response relationships, therapeutic timing, and comparative effectiveness versus existing standard-of-care therapies remain undefined. Addressing these gaps will be essential for determining the true clinical value of CPM in both allergic and antiviral applications (Rizvi et al., 2024; Torres et al., 2021).

Looking ahead, future research should emphasize stereospecific pharmacokinetic assessments to clarify enantiomer-specific exposure differences; the establishment of harmonized PK/PD endpoints to enable cross-study comparisons; rigorous head-to-head evaluations of emerging formulation platforms; and the development of transporter-modulating or mucoadhesive delivery systems capable of enhancing mucosal retention and absorption. Population pharmacokinetic modeling may further help refine dosing recommendations and improve individualized therapy.

## 7 Conclusion

The pharmacokinetics and pharmacodynamics of chlorpheniramine maleate (CPM) demonstrate marked interspecies and formulation-dependent variability. Preclinical studies in rabbits, horses, dogs, cows, and rats consistently highlight differences in ab-sorption rates, bioavailability, and half-life across species. These findings underscore the challenges of extrapolating animal data directly to humans and emphasize the need for stereospecific evaluation of CPM.

In humans, oral and buccal formulations achieve modest systemic exposure with variable bioavailability, whereas intranasal delivery emerges as the most promising route, achieving rapid mucosal absorption and favorable clinical outcomes. Recent trials of intranasal CPM in COVID-19 demonstrated improved symptom control and viral clearance, with complementary observational and pooled evidence suggesting benefits in mitigating long COVID.

Taken together, these findings position intranasal CPM as a candidate for early therapeutic intervention in respiratory illnesses, bridging its historical antihistamine role with potential new applications in infectious disease. Further large-scale, well-designed clinical trials are warranted to confirm these promising results and to establish standardized pharmacokinetic and pharmacodynamic profiles across diverse populations.
